# Improvement of Dielectric, Magnetic and Thermal Properties of OPEFB Fibre–Polycaprolactone Composite by Adding Ni–Zn Ferrite

**DOI:** 10.3390/polym9020012

**Published:** 2017-02-08

**Authors:** Ahmad F. Ahmad, Zulkifly Abbas, Suzan J. Obaiys, Daw M. Abdalhadi

**Affiliations:** 1 Functional Devices Laboratory (FDL), Institute for Advance Material, Universiti Putra Malaysia, 43400 Serdang, Selangor, Malaysia; 2 Department of Physics, Faculty of Science, Universiti Putra Malaysia, 43400 Serdang, Selangor, Malaysia; za@upm.edu.my (Z.A.); diobnsahle@gmail.com (D.M.A.); 3 School of Mathematical & Computer Sciences, Heriot-Watt University Malaysia, 62200 Putrajaya, Malaysia; s.obaiys@hw.ac.uk

**Keywords:** microwave, rectangular waveguide, composites, FTIR, SEM, TGA

## Abstract

The dielectric and magnetic behaviour and thermal properties of composites based on nickel–zinc ferrite (NZF) filler can be improved by the addition of various types of materials. Amongst others, ferrite–polymer composites have been subjected to a wide range of research, due to their extensive applications: electromagnetic interference shielding, microwave absorption, electrodes and sensors. Currently, the interest in scientific and technical searches for the potential outcomes of ferrite–polymer materials due to their different uses in applications such as telecommunication applications, microwave devices and electromagnetic interference shielding has been growing stronger. The dielectric and magnetic behaviour and thermal properties for such composite materials depend on size, shape and the amount of filler addition. Nickel–zinc ferrite material was prepared using the conventional solid-state reaction technique. This study highlights the development of microwave-absorbing material from NZF by adding natural fibres, Oil Palm Empty Fruit Bunch (OPEFB) and polycaprolactone (PCL). OPEFB is considered in this study because it is a solid waste product of the oil palm milling process which is widely and cheaply available. The use of OPEFB in this product may save the environment from oil palm solid waste. A Thermal Hake blending machine was used in blending the powder structure of NZF + OPEFB + PCL, which made it homogeneous. These composites were characterized by the use of Fourier transform infrared (FTIR) spectrometry and scanning electron microscopy (SEM). The thermal degradation behaviour of the composites was analyzed using thermogravimetric analysis (TGA) and differential thermogravimetric (DTG) thermograms. The effective permittivity and effective permeability was obtained over a broad frequency range from 8 to 12 GHz at room temperature. It was observed that the values of effective permittivity and permeability increased as the content of NZF content increased. A rectangular waveguide connected to a microwave vector network analyser (PNA) (HP/Agilent model PNA E8364B) was employed in measuring the reflection coefficient S_11_ and transmission coefficient S_21_ parameters of composites for different percentages of NZF filler. This parameter was then used in calculating the microwave absorbing properties (dB).

## 1. Introduction

Organic–inorganic composites with an organized structure have been extensively studied because they combine the advantages of the inorganic materials (electrical, mechanical strength, thermal stability and magnetic properties) and the organic polymers (dielectric, processability, Flexibility and ductility) which are difficult to obtain from individual components [1].

In designing an electromagnetic absorber, it is important to choose materials that have good control over some magnetic and dielectric properties. Both the magnetic and dielectric properties of the materials must correspond with the frequency of the radiations where a broadband of frequencies must be covered. The materials must be designed in such a way that their magnetic and dielectric properties vary in frequency in a unique fashion [2]. In fact, there are four electromagnetic parameters (the real and imaginary parts of the effective permittivity and magnetic permeability) that must be independently manipulated [3]. Magnetic absorption materials which are made by dispersing magnetic fillers in an insulating matrix continue to play a leading role in the investigation and application of microwave absorption materials [4]. An example of these materials which many researchers have studied, is carbon black polymer composites because their performance is excellent in condensed matter physics as well as in engineering applications such as electromagnetic interference shielding wave absorption, electronic packaging [5,6]. Low amount of Carbon nanotubes (CNTs) nano-sized inclusion was found inside a soft matrix that can largely improve the electrical, mechanical, effective permittivity and effective permeability as well as the thermal properties of the composites in the microwave range of frequencies [7,8].

Ferrite–fibre–polymer composites have several pros, such as easy fabrication into complex shapes, resilience with the required mechanical strength, cost effectiveness, stability, and a wide range of technological applications where numerous magnetic and electrical properties can be observed based on the composition [9]. Scientists and engineers have paid more attention to magnetic polymer composites, where they identify scope for possible exploration for commercial applications in various fields such as electromagnetic interference (EMI) shielding, electrodes and sensors, and microwave absorption [10]. Ni–Zn ferrite is one of the most versatile magnetic material which comes under a group of technologically important magnetic materials based on their distinctive properties such as stability, very good dielectric material, electrical resistivity, low dielectric loss, chemical stability and others which play important roles in the scientific and industrial sectors [11]. Thus, such composites have extensive applications in microwave devices and core materials for power transformers in electronics and telecommunication [12]. Both electrical and magnetic properties of nickel–zinc ferrite (NZF) material strongly depend on the purity of ferrite powder, its microstructure, grain boundary and the chemistry preparation [13].

Polycaprolactone (PCL) was selected in this work because of its high level of flexibility, its previous use as a plasticizer, attractive mechanical properties such as lightweight, flexible, good dielectric properties and ability to be easily fabricated and blended. PCL can be prepared using ring-opening polymerization (ROP) of ε-caprolactone which provides a semi-crystalline PCL-polymer of −60 °C glass temperature with a low melting point between the range of 30–70 °C based on its molecular weight [14,15]. Engineers and researchers have been attracted to using natural fibres for industrial applications because the Oil Palm Empty Fruit Bunch (OPEFB) fibres have a renewable and biodegradable nature with low energy consumption during processing and they are also eco-friendly and cheaper than man-made fibres. The major source of OPEFB is the empty fruit bunch of oil palm trees which is obtained after oil has been extracted from the fruit [16].

The scope of the current study includes the preparation of Ni_0.5_Zn_0.5_Fe_2_O_4_ and its incorporation into an oil palm empty fruit bunch. These materials are a reinforced polycaprolactone matrix used for the improvement of Dielectric, Magnetic and Thermal Properties of the resulting composite. Agilent Technologies 85071E Materials Measurement software was utilized for the evaluation of the effective permittivity and effective permeability of materials and composites under test.

## 2. Experimental Section

### 2.1. Materials

The following were used in the preparation of samples used in this study: OPEFB fibre which was obtained from Ulu Langat Oil Palm Mill, Dengkil, Selangor, Malaysia. Polycaprolactone PCL (C_6_H_10_O_2_) with 97.0% purity and density of 1.146 g/cm^3^ (Sigma Aldrich, Sarasota, FL, USA) purchased from Nature Works LLC (Minnetonka, MN, USA). ZnO (99.9% purity), NiO (99.7% purity) and Fe_2_O_3_ (99.7% purity) (Sigma Aldrich).

### 2.2. Ni_0.5_Zn_0.5_Fe_2_O_4_ Composite Preparation

NZF composite was prepared using a conventional solid-state reaction method. Application of this method provides high temperature based on the high agglomeration in the samples. This high temperature process produces micro size particles which are inappropriate for nonmaterial. The three starting materials used are NiO (99.7% purity), ZnO (99.9% purity) and Fe_2_O_3_ (99.7%) purity. The materials were then weighed according to the following stoichiometric formula

*x* NiO + (1−*x*) ZnO + Fe_2_O_3_ → Ni*_x_*Zn_1−_*_x_*Fe_2_O_4_ (1)

The dried powder was milled and pre-calcite in air at 900 °C for a period of 10 h. Then, the powder was grinded carefully to ensure homogeneity of the powder particles size [17].

### 2.3. Preparation of NZF + OPEFB + PCL Composites

**Figure 1:**
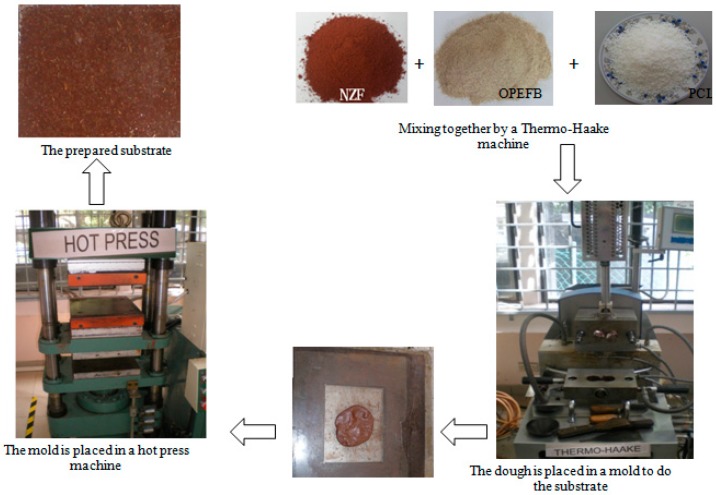
Substrate fabrication steps.

OPEFB-fibre used here was steeped for 24 h in distilled water and then dried in an oven at about 80 °C to remove unwanted materials. In order to eject the wax layer, the filtrated fibre was cleaned out with acetone and then oven-dried again. Afterwards, a grinding device (Mainland, Hunan, China) was used to crush the fibre chains into powder which were sieved neatly to 250 μm size [18]. Afterwards, the OPEFB + PCL composite was mixed carefully alongside NZF filler in a Thermo Haake blending machine polydrive three-phase motor (Motive, Castenedolo, Italy) of 1.5 kW, 3.230 V, 40 A and 50 rpm drive. A total of 40 g sample was prepared for the mixing process. OPEFB + PCL and NZF filler were weighed according to the addition of filler percentage (2.5%–15%) as shown in Table 1. The substrate pellets were prepared by placing 10 g of the blends into a mold of 10 cm × 8 cm dimensions, and the thickness for all the samples was 1 mm due to the difficulty of tightly fitting the sample into the rectangular waveguide to reduce the effect of the air gap between the sample surfaces and the walls of the waveguide. The composite was then preheated for 10 min with an upper and lower plate temperature of 80 °C. A breathing time of 10 s was allowed for the release of bubbles and to reduce void; the composites were then pressed at the same temperature for another 10 min at a pressure of 110 k/bar. The substrate was finally left to cool at room temperature, after which extraction of the sample was performed. The extracted sample was then ready for further characterization. Figure 1 presents the steps used in fabricating the substrates.

### 2.4. Characterisations

#### 2.4.1. Fourier Transform Infrared (FTIR)

FTIR spectroscopy tests were performed to examine the interaction between the fibre, PCL and NZF which were based on the formation of ester bonds at the material interfaces. All analyses were carried out using a Fourier Transform Infrared Spectrometer Perkin Elmer 100 BX (Waltham, MA, USA) equipped with universal attenuated total reflectance. The transmissions of infrared spectra were recorded between 4000 and 400 cm^−1^ frequency ranges.

#### 2.4.2. Thermogravimetric Analysis (TGA) and Differential Thermogravimetric (DTG) Curves

The thermal degradation characteristics of the samples were analysed under flowing nitrogen atmosphere while the regression of samples was carried out under oxidising atmosphere (flowing dry air) at a 200 mL/min flow rate using a (TGA-SDTA 8510) thermo-gravimetric analyser METTLER-TOLEDO (Setaram Instrumentation, Caluire, France). Samples of 9–10 mg each were heated using a temperature range of 20–900 °C at a heating rate of 10 °C/min.

#### 2.4.3. Morphology

The morphology of fracture surface of the composites was observed using a Scanning Electron Microscope (SEM) S-3400N (Yuseong, Daejeon, Korea) at room temperature, with a field emission gun and an accelerating voltage of 10 kV. A gold coating of a few nm thicknesses was placed on the fracture surfaces. The samples were viewed perpendicular to the fractured surface.

#### 2.4.4. Variation in S-Parameter Coefficients with Frequency

The PNA-L based rectangular waveguide measurement mode was utilized in measuring the S-parameters of the two-port network formed by placing the sample under study between two waveguides. The accuracy of the constitutive material properties depended on the accuracy with which the S-parameters were measured. The through-reflect line (TRL) calibration method was used to eliminate systematic errors occurring in the measurement. Since the experimental setup involved many components, such as cables and connectors, proper attention was taken to ensure that the entire system remained stable over the measurement period.

#### 2.4.5. Effective (Relative) Permittivity and Effective Magnetic Permeability of NZF Composites

The material properties of (Ni_0.5_Zn_0.5_Fe_2_O_4_ and NZF + OPEFB + PCL) were studied in the X-band (8–12 GHz) region of the microwave frequency spectra. The dielectric properties (ε′ and ε″) of the sample were measured using a microwave vector network analyser (PNA) (HP/Agilent model PNA E8364B (Agilent Technologies, Santa Rosa, CA, USA) connected to an open-ended coaxial line (probe) by a cable which was attached to the holder and used as a sensor in the measurement of the dielectric properties. The samples were placed flat on the surface of the probe and the measurement technique was based on the reflection coefficient values of the coaxial line versus the composite samples. The effective (relative) permittivity and loss measurements of the samples were taken at a temperature of 27 °C, and then the effective (relative) permittivity ε_r_(*f*) was calculated as:

ε_r_(*f*) = ε′(*f*) − *j*ε″(*f*) (2)

where ε′(*f*) is the effective (relative) permittivity and ε″(*f*) represents the imaginary part of the relative permittivity.

The real and imaginary parts of the effective magnetic permeability were measured using material measurement software Agilent 85071 (Agilent Technologies, Santa Rosa, CA, USA) with a vector network analyser while the effective permeability μ_r_(*f*) was calculated as follows:

μ_r_(*f*) = μ′(*f*) − *j*μ″(*f*) (3)

where μ′(*f*) is the real and μ″(*f*) imaginary parts of effective magnetic permeability of the materials. The inner dimensions of the waveguide are 22.05 mm × 9.27 mm, while the sample dimensions were matched as closely to the waveguide dimensions as possible when placed in the measurement system. Furthermore, great efforts were made by the researchers to ensure that there was no air gap at the corners and that the sample was absolutely flat and flushed against the waveguide.

## 3. Results and Discussion

### 3.1. Fourier Transform Infrared (FTIR)

**Figure 2:**
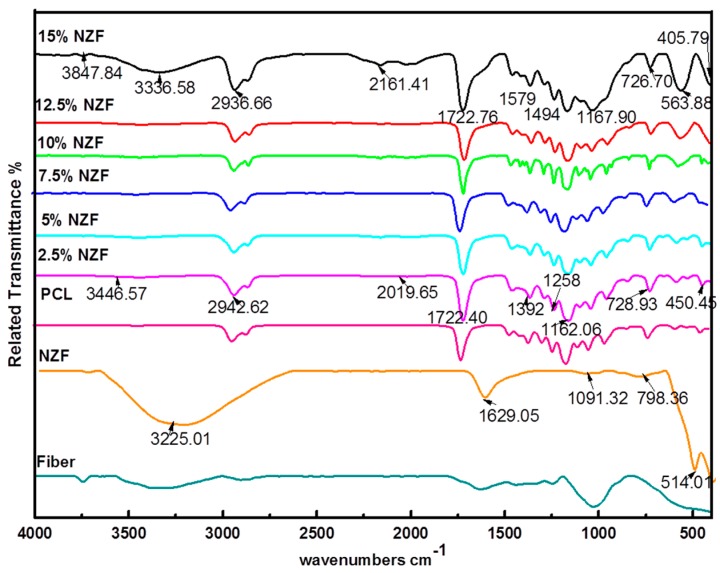
FTIR spectra of OPEFB fibres, NZF, polycaprolactone (PCL) and NZF + OPEFB + PCL for different percentages of NZF filler in the composites.

FTIR spectroscopy is a technique that is sensitive to specific intermolecular interaction [19]. In this study, FTIR analysis has been utilized in investigating the interaction of NZF filler with the OPEFB and PCL matrix. This is done in order to monitor the absorption peak shifts in specific regions and to determine the known functional group interactions. Figure 2 illustrates the FTIR spectrum for pure material PCL, NZF and OPEFB as well as NZF + OPEFB + PCL composites. PCL polymer shows the characteristic peaks at 450.45, 728.93, 1162.06, 1722.40, 2019.65, 2942.62 and 3446.57 cm^−1^. The FTIR spectrum for the pure NZF shows the characteristic peaks at 514.01, 798.36, 1091.32, 1629.05, 3225.01 cm^−1^, demonstrating the purity of PCL and NZF at room temperature. Figure 2 presents the spectra of the composites where the carbon–oxygen (–O–C–O–) stretching bonds give rise to intense and complex multiple peaks in the region between 900 and 1300 cm^−1^ with no extra interaction or new bond formed with the –O–C–O– group. Stretching vibration of –O–C–O– occurred at 1250 cm^−1^ in 2.5% NZF spectrum, which continues to shift to higher energy levels as the percentage of the filler increases.

According to the FTIR spectra of the NZF + OPEFB + PCL composites, the interaction between NZF filler and OPEFB + PCL composites at generality percentages (2.5% to 12.5%) of NZF filler, except for the highest percentage 15% of NZF filler, could be a physical interaction as there is no new beak or any major shift compared to the PCL spectrum. Moreover, after adding more NZF, the enhanced physical properties of NZF + OPEFB + PCL composites could be a result of the entanglement of the NZF and fibre particles–polymer matrix which enhanced the NZF + OPEFB fibre–PCL matrix interfacial strength. This behaviour means that the addition of NZF to the composites improved the interaction between NZF and OPEFB + PCL composites which is evident in the hydrogen–oxygen and carbon–oxygen groups, such as C=O, –O–C–O– and C–O. The peak appearing between 1300 and 1500 cm^−1^ is usually used in characterising a methyl group.

### 3.2. Thermogravimetric Analysis of Composites

**Figure 3:**
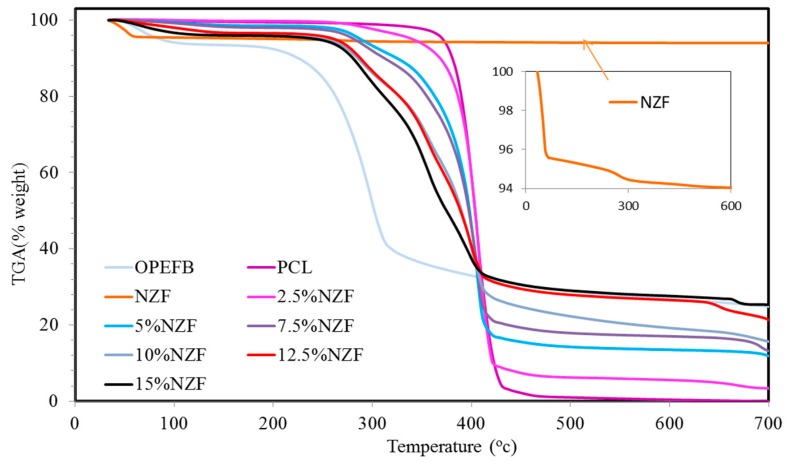
TGA thermograms of OPEFB fibres, NZF, PCL and NZF + OPEFB + PCL for different percentages of NZF filler in the composites.

**Figure 4:**
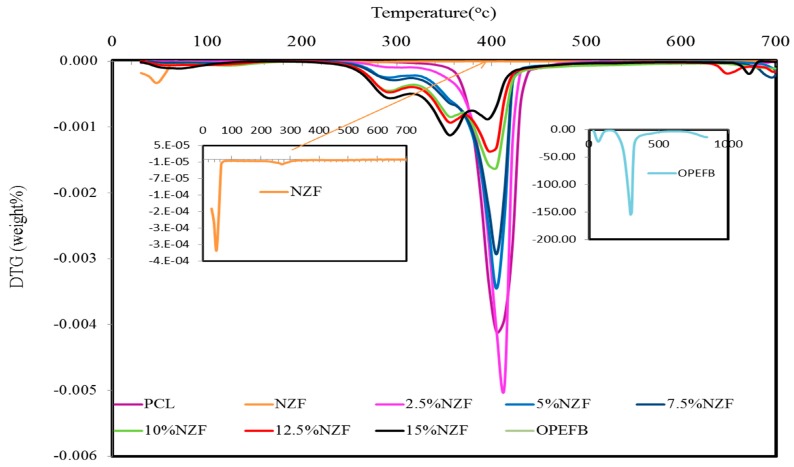
DTG thermograms of OPEFB fibres, NZF, PCL and NZF + OPEFB + PCL for different percentages of NZF filler in the composites.

The thermal degradation characteristics of NZF + OPEFB + PCL are displayed in Figure 3 and Figure 4 by thermogravimetric (TGA) and differential thermogravimetric curves (DTG), respectively. The thermal degradation of OPEFB fibres was a result of the decomposition of cellulose, lignin and hemicelluloses, giving off evaporation [20]. At around 400 °C, the decomposition of the PCL matrix begins while its mass loss of one step degradation procedure starts at about 390 °C and continues very slowly until up to 440 °C. This progression occurs rapidly.

The amount of PCL residue is about 2.1% because of its further breakdown at high temperatures. A second degradation is noticed for the PCL for the thermal degradation of OPEFB + PCL composites with different PCL contents that occur in a two-step degradation process. However, in regards to the decomposition of OPEFB fibres, the composites exhibited an initial mass loss from approximately 200 to 270 °C.

Based on observation, it was found that the second step of the thermal degradation process which is mainly related to the PCL degradation overlaps with cellulose and lignin content in OPEFB. Thus, the two-step degradation process proves that the thermal degradation temperature of the PCL is higher than that of the OPEFB fibres. The NZF content did not degrade in the composites as shown in the thermograms. Generally, the TGA characterisation in Figure 3 shows that the thermal stability of PCL + OPEFB composite was improved after the addition of NZF filler.

To compare the influence of temperature variation on the rate of weight loss for various NZF filler contents on OPEFB + PCL composites, a derivative thermogravimetric (DTG) was performed. From the data presented in Table 2, we can conclude that the values of degradation temperature suggest again some kind of interaction between the PCL matrix and NZF + OPEFB. Based on the weight percentage (wt %) of NZF filler content, the thermal stability of NZF + OPEFB + PCL composites follows the sequence of 15% > 12.5% > 10% > 7.5% > 5% > 2.5%.

### 3.3. Morphological Study of NZF + OPEFB + PCL Composites for Various NZF Filler Contents

**Figure 5:**
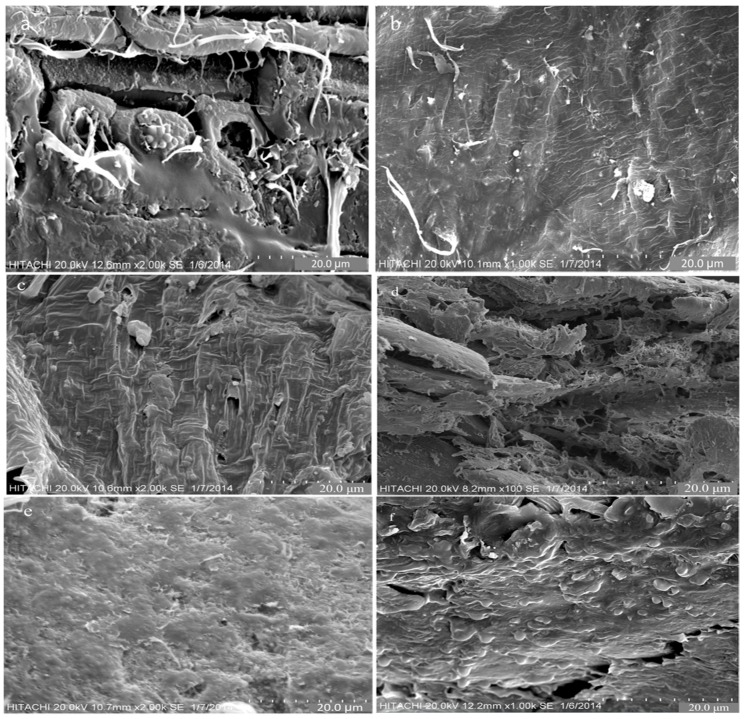
Scanning electron microscopy (SEM) micrographs of NZF + OPEFB + PCL for different percentages of NZF filler in the composites: (**a**) 2.5% NZF; (**b**) 5% NZF; (**c**) 7.5% NZF; (**d**) 10% NZF; (**e**) 12.5% NZF; (**f**) 15% NZF.

Scanning electron microscopy was used to study the surface morphology of the composite materials with NZF as filler material which is based on OPEFB + PCL as the matrix for different percentages of NZF 2.5%, 5%, 7.5%, 10%, 12.5% and 15%. Figure 5a–d illustrates a scanning electron micrograph of the tensile fracture surface of NZF + OPEFB + PCL composites at 2.5%, 5%, 7.5%, and 10% (wt %) of NZF content. At first sight, the dispersion of NZF in the OPEFB + PCL matrix proves the efficient mixing of NZF in the matrix via melt blending of fibres and the PCL polymer in a Thermo Haake blending machine. It is observed that the NZF particles in composites were randomly arranged with weak adhesion of NZF + OPEFB + PCL; the composite showed some gaps and signs of pullout. Figure 5e,f illustrates the effect of NZF 12.5% and 15% on the fracture surface of NZF + OPEFB + PCL composites. It was observed that percentages increased the fracture surface bonding. Moreover, addition of 12.5% and 15% NZF decreased fibre pullout and voids around the fibres by increasing fibre–matrix adhesion. Figure 5 illustrates that as the filler percentage increases in the composites, the adhesion to the polymer + fibre also increases. The pulling of voids and fibre off the composites would have increased energy dissipation during the fracture of composites. Therefore, the result indicates that the NZF particles are well dispersed in the OPEFB + PCL composites. This can be clearly seen with the high concentration of the NZF fillers, especially at 15 wt % in Figure 5f. The imaging further showed that there was strong bonding between the NZF, OPEFB fibre and PCL polymer. The adhesion between all elements was strong. Moreover, the surface of the fibres seemed to be covered with a smooth layer of the filler and matrix. However, voids were still found around the fibre particles through the composites.

### 3.4. Effective Permittivity of NZF + OPEFB + PCL Composites

**Figure 6:**
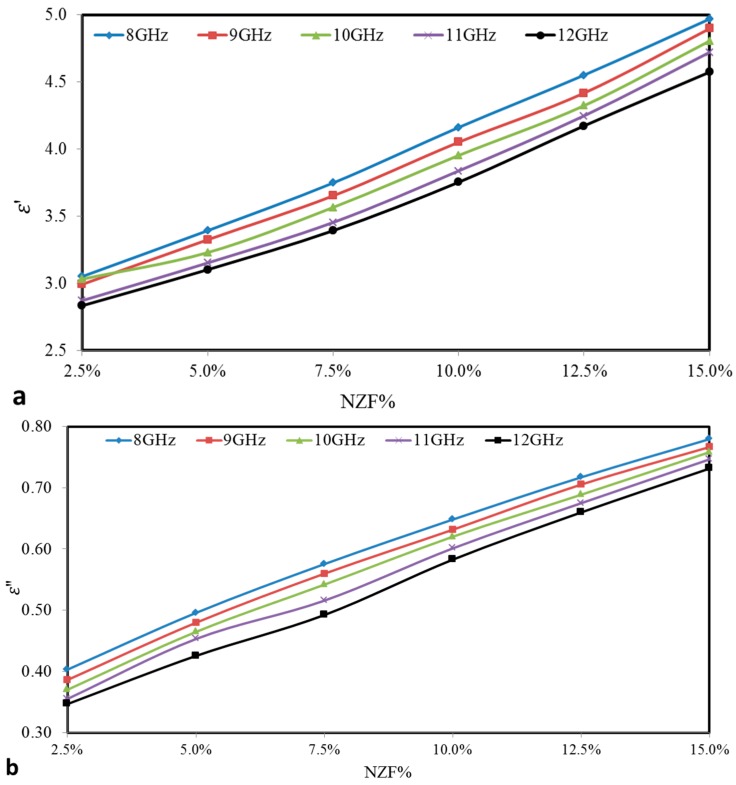
Variations of (**a**) Effective (relative) permittivity (ε′) (**b**) Imaginary part of the relative permittivity (ε″) with different NZF% for NZF + OPEFB + PCL composites.

To investigate the possible mechanism of microwave absorption, the real and imaginary parts of effective permittivity (ε′, ε″) and effective magnetic permeability (μ′, μ″) were determined from the scattering parameters with the help of Agilent Technologies 85071E Materials Measurement software. The real part of the effective permittivity (ε′) is a measure of how much energy from an external electric field is stored in a material. While, the imaginary part of effective permittivity (ε″) is known as the imaginary part of the relative permittivity and is a measure of how much energy is dissipated or lost. Figure 6a,b presents the variation of ε′ and ε″ with loading weight percentage of NZF filler in the composition at 27 °C (room temperature) and random frequencies (8, 9, 10, 11 and 12) GHz. The effective medium theory clearly explains the increment of effective (relative) permittivity and the imaginary part of the relative permittivity values in accordance to the loading filler addition [21,22,23], where a higher effective (relative) permittivity of the polymer-based composite can be obtained by adding high effective (relative) permittivity filler into the low effective (relative) permittivity polymer matrices and vice versa. It is obvious that the values of ε′ and ε″ increase with the increase of ferrite percentage in the composites. Simply, the relation can be described by the following inequalities $\varepsilon_{15\%}^{\prime }>\varepsilon_{12.5\%}^{\prime }>\varepsilon_{10\%}^{\prime }>\varepsilon_{7.5\%}^{\prime }>\varepsilon_{5\%}^{\prime }>\varepsilon_{2.5\%}^{\prime }$ and $\varepsilon_{15\%}^{\prime \prime }>\varepsilon_{12.5\%}^{\prime \prime }>\varepsilon_{10\%}^{\prime \prime }>\varepsilon_{7.5\%}^{\prime \prime }>\varepsilon_{5\%}^{\prime \prime }>\varepsilon_{2.5\%}^{\prime \prime }$. The effective (relative) permittivity and the imaginary part of the relative permittivity at (8 GHz) are the highest compared with other frequencies (9, 10, 11 and 12) GHz. This behaviour is caused by the movement of portable chargers at low frequency which is unstable and reflects dielectric loss.

**Figure 7:**
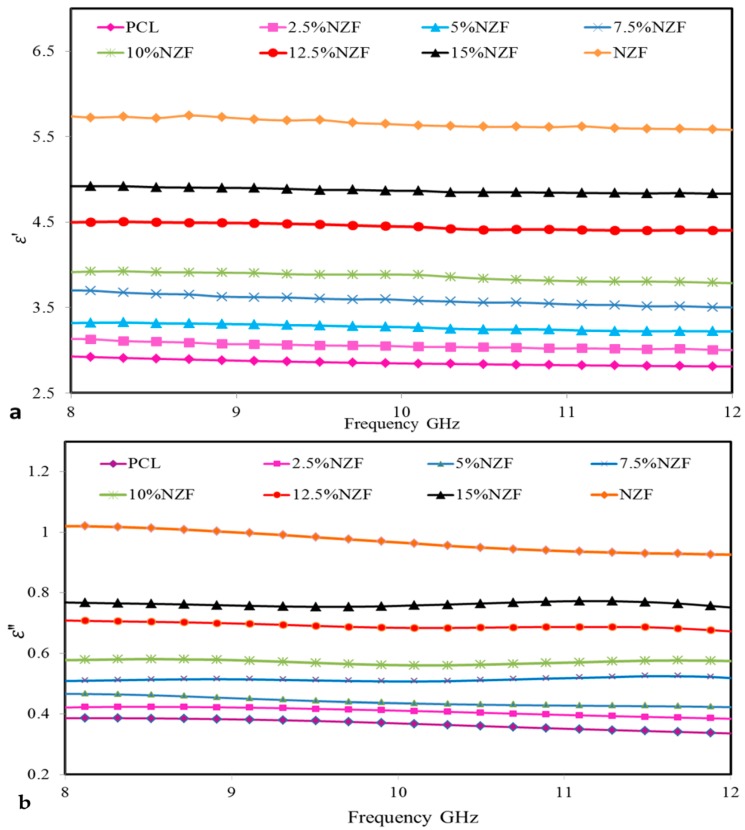
Variation in (**a**) Effective (relative) permittivity ε′ (**b**) Imaginary part of the relative permittivity ε″ with frequency for NZF + OPEFB + PCL composites.

Figure 7a,b shows the variation in dielectric properties of pure NZF, PCL and NZF + PCL + OPEFB composite. It can be observed that NZF exhibits the highest effective (relative) permittivity and imaginary part of the relative permittivity while PCL exhibits the lowest effective (relative) permittivity and imaginary part of the relative permittivity. In Figure 7a, it can be clearly observed that the effective (relative) permittivity of all composites decreases as frequency increases. This may be attributed to space charge polarization. It is suggested that NZF consists of well conducting grains separated by thin insulating grain boundaries [24]. This causes the localized accumulation of charges under the applied external field, thereby enhancing the space charge polarization. Hence, effective (relative) permittivity is expected to be higher in values at low frequencies. At high frequencies, space charge carriers cannot line up their axes parallel to the field, thereby reducing the contribution of space charge polarization. Thus, effective (relative) permittivity is expected to be lower at high frequencies. Similar behaviour was also reported for other fibre reinforced polyesters [25].

**Figure 8:**
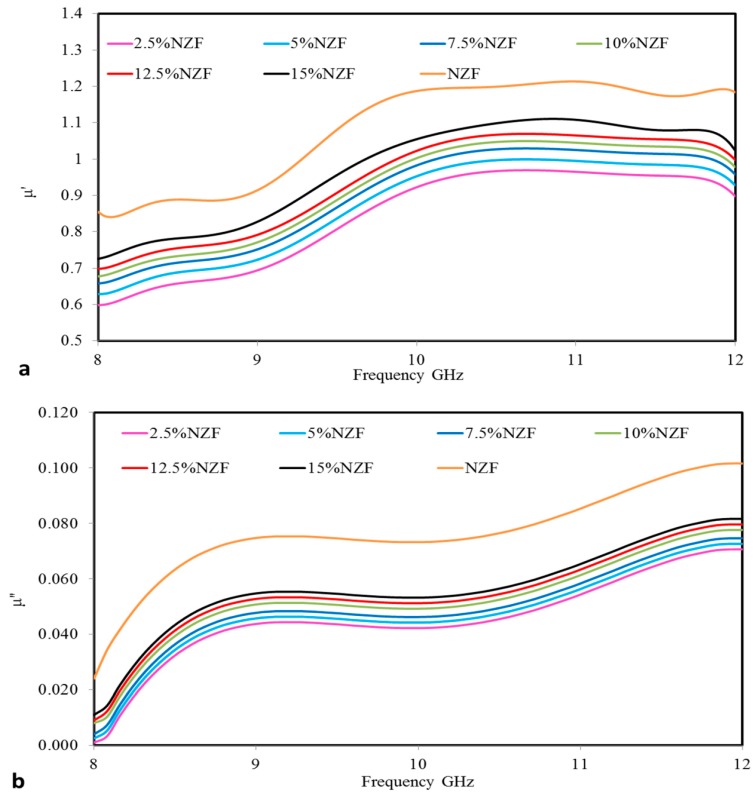
Frequency dependence of (**a**) real part of the effective magnetic permeability (μ′) and (**b**) imaginary part of effective magnetic permeability (μ″) for NZF + OPEFB + PCL composites.

The variation of ε″ with increasing frequency for NZF + OPEFB + PCL composites is shown in Figure 8b. It can be observed that the imaginary part of the relative permittivity decreases with increasing frequency. This might be due to the fact that more energy is needed for the hopping process in the low frequency region which corresponds to high resistivity. Table 3 shows the real and imaginary effective permittivity measurements at a frequency of 10 GHz for all samples. 

### 3.5. Effective Permeability of NZF Composites

The elements which are contained in NZF explain the mechanism of the effective magnetic permeability for NZF. It has an inverse spinel structure in which Ni^2+^ and half of Fe^3+^ ions occupy the octahedral hole, while Zn^2+^ and rest of the Fe^3+^ ions occupy the tetrahedral hole. Interfacial polarization may occur at the interface between PCL and NZF. Interfacial polarization may also occur between OPEFB + PCL and NZF. The electric field loss is caused by the dielectric relaxation effect associated with permanent and induced molecular dipoles. An applied electric field creates a torque on the electric dipole and the dipole rotates to align with the electric field i.e., there is an occurrence of orientation polarization. At microwave frequencies i.e., at higher frequencies, the electric field energy changes quickly. There occurs a lack of alignment due to homogeneity of the composite material [26]. The real (μ′) and imaginary (μ″) parts of the effective magnetic permeability measurements of pure Ni_0.5_Zn_0.5_Fe_2_O_4_ and NZF + OPEFB + PCL composites vs. frequency are shown in Figure 8a,b. The pure Ni_0.5_Z_0.5_Fe_2_O_4_ sample shows μ′ values of around 0.846 at 8 GHz, increasing up to 1.216 at 10.64 GHz and thereafter decreasing to 1.185 at 12 GHz. Meanwhile, μ″ values present a low magnetic loss value of around 0.024 at 8 GHz, then increasing to 0.1016 at the end of the frequency range (12 GHz), together with the effective permeability values for all the samples which behave similarly with pure NZF.

The NZF + OPEFB + PCL composites with different percentages of filler NZF (2.5 wt %, 5 wt %, 7.5 wt %, 10 wt %, 12.5 wt % and 15 wt %) vs. frequency show that μ′ values start (0.596, 0.626, 0.656, 0.676, 0.696 and 0.726) at 8 GHz while the μ″ values start (0.001, 0.003, 0.004, 0.008, 0.009 and 0.011) respectively. The trend of the effective magnetic permeability curves shows an increment where the NZF filler increases due to the interactions of the spin and domain wall with the magnetic fields. Frugally, μ′ and μ″ of the composites at different NZF% filler can be described by the following inequalities $\mu_{15\%}^{\prime }>\mu_{12.5\%}^{\prime }>\mu_{10\%}^{\prime }>\mu_{7.5\%}^{\prime }>\mu_{5\%}^{\prime }>\mu_{2.5\%\;}^{\prime }\;\mathrm{and}\;\mu_{15\%}^{\prime \prime }>\mu_{12.5\%}^{\prime \prime }>\mu_{10\%}^{\prime \prime }>\mu_{7.5\%}^{\prime \prime }>\mu_{5\%}^{\prime \prime }>\mu_{2.5\%}^{\prime \prime }$ respectively. Table 4 indicates that all μ′ and μ″ values increase regularly as the filler percentage of the composite increases at a fixed frequency value (10 GHz).

### 3.6. Absorption Measurement

The material reflection loss (Γ) can be calculated from the magnitude of reflection S_11_ and transmission S_21_ coefficient values as a function of frequency for the samples with a limited thickness of 1 mm using the relation given by [27].

(4) $$\Gamma =x\pm \sqrt{\left(x^{2}+1\right);\;\left|\Gamma \right|\leq 1}$$

Where *x = (S*_11_^2^ − S_21_^2^ + 1)/2S_11_ (5)

Reflection Loss (dB) = −20log [Γ](6)

**Figure 9:**
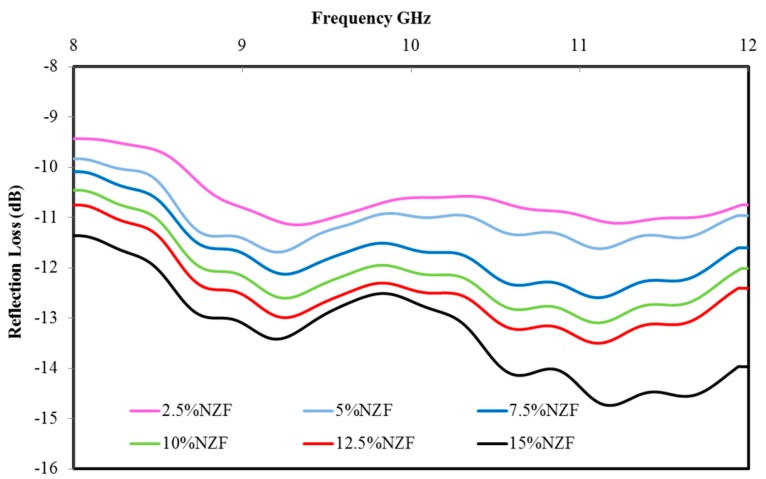
Variation in absorption of NZF + OPEFB + PCL composites.

Figure 9 represents the measured absorption spectra of (2.5%, 5%, 7.5%, 10%, 12.5% and 15%) NZF filler. The prepared samples showed the absorbing properties in a wide frequency range in the (8–12 GHz) X-band region. All samples showed the maximum reflection loss of −11.12 dB at 9.26 GHz, −11.68 dB at 9.22 GHz, −12.58 dB at 11.1 GHz, −13.09 dB at 11.1 GHz, −13.49 dB at 11.1 GHz and −14.73 dB at 11.01 GHz, respectively. A total of 15% NZF showed the maximum reflection loss, i.e., better absorption than the other percentages of filler. As the concentration of NZF decreases, the absorbing properties decreased. Increasing the NZF% values in the composites resulted in higher conductivity outcomes that contributed to the higher effective permittivity results. The mechanism of microwave absorption was clearly described with the help of real and imaginary parts of effective permittivity and effective magnetic permeability.

The values of reflection loss for the different substrate NZF percentages at 8, 9, 10, 11 and 12 GHz are presented in Table 5. Careful observation of selected frequencies in Table 5 reveals that an increase occurs in absorption coefficients as the sample percentage increases. However, the existence of NZF may take part in compensating the dipole moment of PCL [28]. Thus, the lowest percentage of filler and absorption value is the absorption value of 2.5 wt % of NZF composite which is the lowest compared to others. Percentage of filler and the absorption value of 15 wt % of NZF composites is the highest, as clearly indicated in the table.

## 4. Conclusions

Based on the results obtained from the experiments, the following conclusions can be drawn:

Ni_0.5_Zn_0.5_Fe_2_O_4_ composites were successfully prepared based on the conventional solid-state reaction technique. A powder mixture of the samples was milled and blended using a Thermo Haake machine. The performance of the components with different ratios of NZF + OPEFB + PCL in the frequency range 8–12 GHz was discussed. The characterisations of the prepared materials were done using FTIR, TGA and DTG analyses which showed that NZF fillers had less thermic stability than the polycaprolactone matrix alone; this may be attributed to the polymer chain breakages that occurred in the composite matrix as a result of extrusion. The surface morphology was studied using SEM. The dielectric behaviour of the NZF + OPEFB + PCL samples that were measured through an open-ended coaxial probe revealed a proportional relation between the obtained results of ε′ and ε″ with NZF filler percentage and reacting inversely with the elevated frequency. Simply, the relation can be described by the following inequalities $\varepsilon_{15\%}^{\prime }>\varepsilon_{12.5\%}^{\prime }>\varepsilon_{10\%}^{\prime }>\varepsilon_{7.5\%}^{\prime }>\varepsilon_{5\%}^{\prime }>\varepsilon_{2.5\%}^{\prime }$ and $\varepsilon_{15\%}^{\prime \prime }>\varepsilon_{12.5\%}^{\prime \prime }>\varepsilon_{10\%}^{\prime \prime }>\varepsilon_{7.5\%}^{\prime \prime }>\varepsilon_{5\%}^{\prime \prime }>\varepsilon_{2.5\%}^{\prime \prime }$. Furthermore, both μ′ and μ″ parts of the effective magnetic permeability were successfully measured as a function of frequency using the material measurement software Agilent 85071E with a PNA. All values of μ′ and μ″ increased gradually due to the increased filler percentage and frequency. Clearly, the relation can be described by the following inequalities $\mu_{15\%}^{\prime }>\mu_{12.5\%}^{\prime }>\mu_{10\%}^{\prime }>\mu_{7.5\%}^{\prime }>\mu_{5\%}^{\prime }>\mu_{2.5\%}^{\prime }$ and $\mu_{15\%}^{\prime \prime }>\mu_{12.5\%}^{\prime \prime }>\mu_{10\%}^{\prime \prime }>\mu_{7.5\%}^{\prime \prime }>\mu_{5\%}^{\prime \prime }>\mu_{2.5\%}^{\prime \prime }$. The composite sample of 15% filler showed the maximum reflection loss of (−14.37) dB at 11 GHz. Moreover, 2.5% NZF filler of the material showed the lowest Γ of (−9.43) dB at 8 GHz.

## Figures and Tables

**Table 1:** Percentage of materials in the composite.

Percentage of NZF wt %	Percentage of OPEFB wt %	Percentage of PCL wt %
2.5	12.2	85.3
5	23.8	71.2
7.5	34.7	57.8
10	45	45
12.5	54.7	32.8
15	63.8	21.2

**Table 2:** Summary of DTG degradation temperatures of NZF + OPEFB + PCL composites with various NZF wt % contents.

Filler (%)	First peak (°C)	Second peak (°C)	Third peak (°C)	Fourth peak (°C)	Fifth peak (°C)
Pure NZF	51.51	285.80	-	-	-
2.5	-	-	-	410.50	667.25
5.0	-	283.61	-	406.50	-
7.5	-	-	-	405.94	692.79
10.0	121.74	286.27	354.37	403.69	698.03
12.5	61.17	287.74	354.74	402.10	649.22
15.0	51.51	285.80	-	-	-

**Table 3:** Variations in ε′ and ε″ values at 10 GHz.

Percentage of NZF wt %	ε′	ε″
100% PCL	2.844	0.367
2.5%	3.058	0.390
5%	3.257	0.434
7.5%	3.592	0.506
10%	3.856	0.560
12.5%	4.446	0.683
15%	4.868	0.758
100% NZF	5.632	0.962

**Table 4:** Variations in μ′ and μ″ values at 10 GHz.

Percentage of NZF wt %	μ′	μ″
2.5	0.92	0.042
5	0.95	0.045
7.5	0.98	0.046
10	1.00	0.048
12.5	1.02	0.051
15	1.05	0.054
Pure NZF	1.17	0.073

**Table 5:** Variation of reflection loss for NZF + OPEFB + PCL composites.

NZF%	8 GHz	9 GHz	10 GHz	11 GHz	12 GHz
2.5%	−9.43	−10.80	−10.61	−10.96	−10.75
5%	−9.83	−11.41	−10.97	−11.51	−10.96
7.5%	−10.09	−11.71	−11.63	−12.50	−11.60
10%	−10.46	−12.16	−12.08	−13.00	−12.01
12.5%	−10.75	−12.53	−12.44	−13.40	−12.41
15%	−11.36	−13.10	−12.66	−14.37	−13.97
